# Household income and retirement perspective among older workers in Germany—Findings from the lidA Cohort Study

**DOI:** 10.1002/1348-9585.12130

**Published:** 2020-05-14

**Authors:** Hans Martin Hasselhorn, Melanie Ebener, Athanasios Vratzias

**Affiliations:** ^1^ Department of Occupational Health Science University of Wuppertal Wuppertal Germany; ^2^ Department of Psychology Aristotle University of Thessaloniki Thessaloniki Greece

**Keywords:** extended working lives, finances, health, motivation to work, social status, work exposure

## Abstract

**Introduction:**

In times of extending working lives, it is relevant to understand why, today, most workers leave employment long before regular retirement age. Financial factors have been central for explaining retirement timing, yet their impact seems rather complicated. This study explores the motivation to keep working, in relation to the economic household conditions among older workers and it investigates the impact of socio‐demographic, individual and work factors on the motivation to keep working (MTW), again differentiated by economic household condition.

**Methods:**

Based on data from wave three (2018) of the lidA Cohort Study, a representative interview study of socially insured employees born in 1959 or 1965 in Germany, descriptives and linear regression analyses were performed among 2835 employed participants. For all analyses, the sample was divided into five household equivalized net income groups.

**Results:**

The groups with low and second lowest income were most motivated to work longer, followed by those with the highest income. The lowest income group exhibited most adverse scores for work indicators and health. Furthermore, in this group, MTW was unrelated to physical and mental health indicating that the comparably high motivation may be driven by financial imperatives.

**Discussion and Conclusions:**

The findings indicate that many workers with low income may find themselves forced to extend their working life, irrespective of their health. This requires increased attention by research as well as policy. Policy might have to acknowledge group specific negative side effects of regulations effectively extending working lives.

## 1 INTRODUCTION

Many modern industrialized countries are witnessing lower birth rates, increasing life expectancy and thus a compositional shift from younger to older age groups. This development is pronounced in Germany, which today has one of the highest old‐age dependency ratios in the European Union and will continue so at least until 2050.^1^ Population ageing has raised concerns regarding the sustainability of the welfare state system and in many countries led to the implementation of reforms aiming at higher work participation of older workers. In Germany, these reforms mainly relate to the reduced access to early retirement and a gradual increase of the statutory retirement age from the age of 65 to 67. These measures have contributed to an increase of the employment rates among older workers, for example from 33% in 2007 to 58% in 2017 among those aged 60‐64 years, which is the second highest rate in the European Union behind Sweden (68%).^2^

In light of these policy changes, it is relevant to understand why, still today, most workers leave employment long before regular retirement age. Some studies and theories suggest that employment participation is determined by the interaction of multiple factors.^3^, ^4^, ^5^, ^6^ The “lidA conceptual model of work, age and employment,” for example, postulates that employment participation at higher working age is a function of the complex interaction of eleven domains: social status, domestic domain, work content, work organization, health related life style, health, work ability, motivation to keep working (MTW), labor market, social context and last, and not least financial factors.^4^, ^7^

Financial factors have been central for explaining the retirement age historically.^7^ Yet, while the impact of some domains on employment participation of older workers seems rather clear‐cut—such as the work content—the impact of finances seems rather complicated.^8^ While authors from many countries show that workers of lower social class who, naturally, have less financial resources, are much more likely to leave employment early (see, for Sweden,^9^ for UK,^10^ for NL^11^), others report the observation that poorer workers are motivated to work longer, namely by financial factors,^12^ and that workers with debts are more likely to expect to stop working later.^13^ This apparent contradictive observation is also evident for those financially well off: In his overview report, Lain^7^ has collected evidence from the UK and further European countries indicating that those with high financial resources are those with the highest probability of retiring early—when health reasons are taken out. Also Damman et al^3^ have found in a study investigating men in the Netherlands that the wealthier the workers, the stronger was their intention to retire early and the earlier they retired—thereby referring to and confirming economic rational choice theory.^3^, ^8^ Yet, at the same time, studies have found that workers of higher socio‐economic status are more intrinsically motivated to work, leading to a higher motivation to keep working^12^ and to a later exit from employment.^7^

The above diversity of findings indicates, that different aspects accompany and potentially modify the impact of financial factors on employment participation at higher working age. To the best of our knowledge, this issue has never been addressed in a larger sample of the older working population in Germany. Now, the German lidA Cohort Study on Work, Age, Health and Work Participation provides the opportunity to investigate the association of finances with the workers’ retirement perspective in a large representative sample of older workers approaching retirement age.

Overall, our assumption is that among older workers—on average—two economic groups exhibit a higher MTW: those with lowest financial resources because of economic dependencies, and those with largest financial resources because of more intrinsically motivating work. In this study, this shall be investigated by taking advantage of a representative sample of older workers in Germany. In a second step, we want to explore the predictors of MTW, namely socio‐demographic, individual and work factors, separately by economic group. It is not the authors’ aim to identify causal relationships in this cross‐sectional study.

## 2 METHODS

### 2.1 Study population

Data from the German lidA Cohort Study on Work, Age, Health and Work participation was used (http://www.lida-studie.de). The study is representative for employed people subject to social security contributions (no self‐employed or sworn civil servants), born in either 1959 or 1965 in Germany.^14^, ^15^ Data collection is based on computer‐assisted personal interviewing (CAPI), which has taken place in 2011 (wave 1, N = 6585), 2014 (wave 2, N = 4244), and 2018 (wave 3, N = 3586) in their homes. A more detailed description of the design of the lidA Cohort Study has been given elsewhere.^1^ The current study considers cross‐sectional data from wave 3. For analysis we have selected participants who were employed full time, part time or marginally, not including unemployed, those exclusively working self‐employed and retirees who were not working. Among the remaining 3270 participants, those were selected for analysis who had no missings for any of the variables included (additional loss of 435 participants, 13.3% of 3270). As a result, the sample consists of 2835 participants. 1585 participants (56%) belonged to the younger age group which reflects deliberate oversampling of younger workers in the study, and 1535 (54%) were women.

### 2.2 Variables used

Household income was inquired by asking for income from twelve different sources (eg individual wage, child allowances). Finally, the participant had to estimate the net household income in sum. To reduce social desirability effects, the answer categories are coded by letters that are presented on a list in a non‐ascending order. This is a procedure commonly performed in large interview studies. Finally, household equalized net income was calculated by dividing the household income by the square root of household size according to OECD Square Root Scale.^16^

The outcome MTW was measured with an own 5‐item scale conceptually based on the understanding of MTW as published by Kanfer et al^17^: “Motivation to work pertains to cognitions, affect, and behaviors related to participation in an observable work arrangement.” The conceptual focus is on the boundaries of work and retirement and contains questions such as, “It is highly likely that I will work up to the legal retirement age” and “I cannot imagine that I will stop working one day”. MTW is computed as the mean score of the five items, theoretically ranging from 1 (low MTW) to 5 (maximum MTW). Response options range from ‘does not apply at all’ to ‘applies fully’. Cronbach's alpha was 0.73.

“Affordability,” “attitude of social environment towards early exit” and “ability to continue working” were self‐reported single items responding to the questions/statements, “I could afford to retire from working life before the statutory retirement age”, “In my personal environment there is an attitude to quit work earlier rather than later” and “And what do you think until what age you can work?”, respectively. Leadership quality was measured with a 3‐item scale from the Copenhagen Psychosocial Questionnaire (COPSOQ‐II, middle version^18^). Work centrality, indicating the current general importance of work in a participants’ life, was assessed using a selection of 3 items from the 5‐item scale by McDonald and Levy.^19^ Physical health (physical component summary) and mental health (mental component summary) were assessed with the Short Form 12 Health Survey.^20^, ^21^

### 2.3 Statistical methods

The sample was divided into five household groups of equivalized net income (<60% of sample mean, up to 80%, up to sample mean, up to 150%, >150%). The group size differed between 324 (lowest income group, 11% of the sample) and 948 (“up to sample mean” income group, 33% of the sample). Across these five income groups, counts of sociodemographic variables (age, gender) and group means of “affordability of early exit”, leadership quality, attitude of social environment towards early exit, ability to continue working, work ability, physical as well as mental health were compared. Mean differences for MTW between the five income groups was assessed by ANOVA with Bonferroni post hoc tests. Significance levels of differences between the five income groups were determined performing multivariate analysis of covariance (MANCOVA) and Chi‐square tests.

In the exploratory analysis, multiple linear regression models were used to better understand the differentiated effect that potential predictors have on MTW, when looking at different net income groups separately. Possible predictors selected for the analyses were age, gender, affordability, leadership quality, attitude of social environment toward early exit, ability to continue working, work ability, physical and mental health. All these variables were included in the models. VIF scores in the collinearity statistics for theH five multivariable models were always <2, therefore multicollinearity was not assumed.

## 3 RESULTS

**FIGURE 1:**
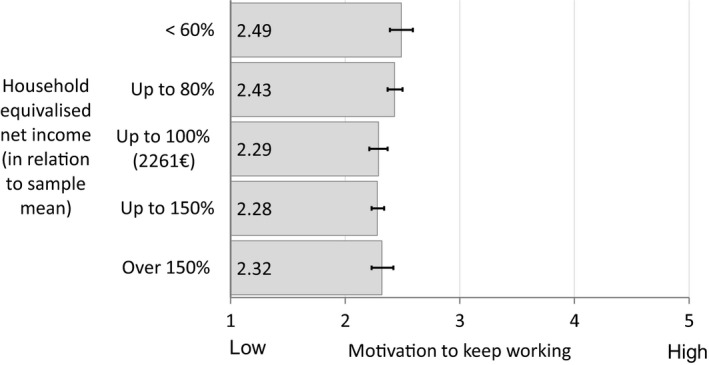
Mean scores for the Motivation to Work Scale by income group (household equivalized net income) among older workers in Germany. Errorbars indicate 95% confidence intervals of the standard error of the mean

Figure 1 depicts that household equivalized net income is associated with MTW in a j‐shaped manner. The overall mean for MTW was 2.35 (SE_Mean_ 0.02). The lowest income group (A) exhibited the highest MTW, followed by the second lowest income group (B). The medium (C) and medium‐high (D) income groups had lowest MTW scores and the highest income group (E) somewhat higher MTW scores. Post hoc testing showed that MTW in group A was significantly lower than MTW in groups C (*P* < .05) and D (*P* < .01), MTW in Group B was significantly lower than MTW in group D (*P* < .05) (Table 1).

**TABLE 1:** Description of the sample, outcome, confounders, and predictors. N = 2835, workers in Germany, born in 1959 or 1965. The sample is representative for the socially insured working population of same age

Household equivalized net income group		(Α) <60% N = 324	(Β) up to 80% N = 694	(C) up to 100% (2261€) N = 535	(D) up to 150% N = 948	(E) over 150% N = 334
Outcome	Range	Mean	Mean	Mean	Mean	Mean
Motivation to work (95% CI)	1‐5 (SE_Mean_=0.02)	2.49_CD_ (2.39‐2.59)	2.43_D_ (2.37‐2.50)	2.29_A_ (2.21‐2.37)	2.28_AB_ (2.23‐2.34)	2.32 (2.23‐2.42)

Confounders		Count (%)	Count	Count	Count	Count
Age*	1959	136 (42.0)	268 (36.6)	246 (46.0)	434 (45.8)	166 (49.7)
	1965	188 (58.0)	426 (61.4)	289 (54.0)	514 (54.2)	168 (50.3)
Gender**	Men	115 (35.5)	297 (42.8)	238 (44.5)	468 (49.4)	182 (54.5)
	Women	209 (64.5)	397 (57.2)	297 (55.5)	480 (50.6)	152 (45.5)

Predictors	Range	Mean	Mean	Mean	Mean	Mean
Affordability^a^ (95% CI)	1‐4	3,33_BCDE_ (3.23‐3.42)	2,96_ADE_ (2.89‐3.04)	2,87_ADE_ (2.79‐2.95)	2,48_ABCE_ (2.42‐2.54)	2,07_ABCD_ (1.97‐2.17)
Early exit: attitude of social environment^b^ (95% CI)	1‐4	2.10_D_ (2.00‐2.21)	1.98 (1.91‐2.05)	1.96 (1.88‐2.04)	1.90_A_ (1.84‐1.95)	1.99 (1.89‐2.08)
Leadership quality^c^ (95% CI)	0‐100	49.2_BE_ (46.6‐51.8)	53.6_A_ (51.8‐55.3)	51.5 (49.6‐53.5)	53.4 (51.9‐54.9)	55.9_A_ (53.4‐58.5)
Ability to continue working (age)ᵈ (95% CI)	Mean age	64.3_DE_ (63.9‐64.7)	64.8_E_ (64.5‐65.0)	65.0_E_ (64.7‐65.3)	65.1_AE_ (64.9‐65.4)	66.2_ABCD_ (65.7‐66.6)
Work centralityᵉ (95% CI)	1‐5	2.40 (2.29‐2.50)	2.30 (2.24‐2.37)	2.33 (2.26‐2.40)	2.32 (2.26‐2.37)	2.36 (2.27‐2.45)
Physical healthᶠ (95% CI)	0‐100	45.3_CDE_ (44.3‐46.3)	46.9_DE_ (46.2‐47.6)	47.3_ADE_ (46.5‐48.1)	49.1_ABCE_ (48.6‐49.7)	51.0_ABCD_ (50.0‐51.9)
Mental health^ᶢ^ (95% CI)	0‐100	49.3_DE_ (48.1‐50.4)	51.2_E_ (50.5‐52.0)	51.0_E_ (50.2‐51.9)	52.2_A_ (51.6‐52.8)	53.7_ABC_ (52.7‐54.6)

Note

Capital letters _A‐E_ indicate significance of difference of the values in this column to the values in the respective column.

^a,b^High scores indicate a low expression of the concept.

^c,d,e,f,g^High scores indicate a high expression of the concept.

* Chi‐square test *P* < .01;

** Chi‐square test *P* < .001.

Table 1 also shows the means or proportions of confounders and of the predictors for all five income groups investigated. The mean score for *work centrality* did not differ significantly between the five income groups. Otherwise, significant group differences were found in all instances, always with the most adverse scores for the lowest income group. To give two examples: *Affordability* showed a pronounced gradient starting from 3.33 (lower affordability) for the lowest income group (A) and ending with 2.07 (high affordability) for the highest income group (E). And the positive *attitude of the social environment towards early exit* was most pronounced among those belonging to the lowest income group (A) and least pronounced among respondents in the highest income group (E).

Table 2 displays results from multiple linear regression analysis stratified by income groups. Overall, the variable sets predicted MTW in all income groups significantly and substantially (*R* ^2^ ranging from 0.31 to 0.39). Age was not a significant predictor of MTW across any income group. Women exhibited higher MTW in all groups. Three of the predictors displayed significant effects on MTW in each of the five income groups: *attitude of the social environment towards early exit* (if attitude was positive towards a longer working life, then MTW was higher), *ability to continue working* (ability to work until higher age was associated with high MTW) and *work centrality* (higher work centrality was associated with higher MTW). The remaining four predictors (affordability, leadership quality and the two health scales) were significantly associated with MTW in some, but not all income groups.

**TABLE 2:** Multiple linear regression analysis for motivation to keep working (MTW score) separately for five household equivalized net income groups. N = 2835, workers in Germany, born in 1959 or 1965. The sample is representative for the socially insured working population of same age. Bold figures indicate significant association of the independent variable with MTW in the respective group

Household equivalized net income group	Range	(Α) <60% N = 324		(Β) up to 80% N = 694		(C) up to 100% (2261€) N = 535		(D) up to 150% N = 948		(E) over 150% N = 334	
		β	*P*	β	*P*	β	*P*	β	*p*	β	*P*
(Constant)			.05		.94		.58		.86		.38
Age	1959, 1965	0.09	.07	−0.01	.78	0.01	.81	−0.01	.86	0.03	.48
Gender^a^	1‐2	**0.12**	**.01**	**0.12**	**.00**	**0.15**	**.00**	**0.09**	**.00**	**0.11**	**.02**
Affordabilityᵇ	1‐4	**0.11**	**.02**	**0.09**	**.00**	0.02	.49	**0.19**	**.00**	**0.17**	**.00**
Early exit: attitude of social environment^c^	1‐4	**0.22**	**.00**	**0.21**	**.00**	**0.22**	**.00**	**0.20**	**.00**	**0.23**	**.00**
Leadership quality^d^	0‐100	0.06	.20	**0.08**	**.01**	**0.07**	**.04**	0.02	.58	0.02	.71
Ability to continue working (age)ᵉ	Mean age	**0.32**	**.00**	**0.36**	**.00**	**0.39**	**.00**	**0.33**	**.00**	**0.27**	**.00**
Work centralityᶠ	1‐5	**0.30**	**.00**	**0.28**	**.00**	**0.27**	**.00**	**0.20**	**.00**	**0.26**	**.00**
Physical healthᶢ	0‐100	0.03	.57	**0.08**	**.02**	0.01	.74	0.05	.08	0.08	.10
Mental healthʰ	0‐100	0.04	.38	**0.10**	**.00**	**0.09**	**.02**	**0.12**	**.00**	**0.12**	**.02**
*R* ^2^		0.358		0.389		0.373		0.312		0.318	

^b,c^High scores indicate a low expression of the concept.

^d,e,f,g,h^High scores indicate a high expression of the concept.

^a^ Gender (1 = men, 2 = women).

The groups exhibited characteristic patterns. The two groups with a household equivalized net income above the mean (D and E) showed identical results: affordability and also mental health revealed strong effects on MTW, but not leadership quality and physical health. In contrast, the middle group (C) was the only income group where affordability was not associated with MTW. Also mental health did not show an effect. In the lower income group B, however, all predictors were strongly associated with MTW. The lowest income group did not show a significant association of leadership quality with MTW, yet the beta score indicates that—compared to groups B and C—the lower number of cases in this group may have contributed to non‐significance. Neither of the two health indictors influenced MTW significantly in this group.

## 4 DISCUSSION

The aim of this explorative paper was to investigate MTW by household income group among older workers in Germany and to understand group differences by assessing the differential impact of predictors in each of the groups. MTW was highest in the lowest two income groups and it was lowest in the middle‐income groups. The absolute differences seem small but they are significant, which is due to the small standard error of the mean characteristic for the MTW scale. Furthermore, the predictors were most adverse for those with the lowest income. All independent variables investigated except age, exhibited significant associations with MTW in at least one of the income groups. Some patterns emerged: according to our findings in high‐income groups (D and E), leadership quality at work does not seem to matter when it comes to MTW. Also, physical health neither matters for those with very low nor for those with higher income. Most sensitive to any predictor seems to be the group with low, but not very low income (B). Finally, health—whether physical or mental—does not influence MTW among those with lowest income (A).

### 4.1 Differential non‐linear impact of finances

Our study confirms that financial status may have a differential impact on the issue of employment participation at higher working age: among those working, the poorest are motivated to work longer. At first sight, this may contradict the observation that the poorest are leaving employment much earlier than those financially well off.^9^, ^10^

Furthermore, our findings are in line with previous observations that the relationship of financial status to employment participation is not linear.^22^, ^23^ The groups with lowest MTW and thus least attached to employment participation were the middle‐income groups, while the highest income group showed a somewhat higher MTW, and the lowest income group the highest MTW. This is in line with a statement by Meadows^12^: “Those who are likely to want to continue working tend to fall into two distinct groups: those who are better qualified, and who have or can obtain intrinsically enjoyable jobs which are not too stressful or challenging, and another, generally poorer, group who are motivated mainly by financial factors.”

### 4.2 Adverse conditions force low‐income workers to employment

Numerous authors report financial hardship forcing the poorest to keep working. Szinovacz, Davey and Martin^13^ for example, found that people with debts expect to work longer. In our study low household income had an impact on the workers’ long‐term perspective on employment career and retirement, indicating an awareness for reduced financial security.^24^ Over and above, our results show that the low‐income group is additionally burdened with adverse exposures on the personal and work level. This makes us speculate, that the accumulation of personal risks among older low‐income workers provides an uncertain individual future not only in respect to finances, but also to health and social participation. In such fragile personal environments and perspectives, continued employment may be key for social participation.

### 4.3 No primacy of finances with respect to retirement timing

Some scientists have questioned the primacy of finances when it comes to retirement timing. Loretto and Vickerstaff^25^ have found finances often being overridden by factors from the domestic domain in their qualitative study. In a review, Wurm et al^26^ quote several studies documenting a stronger effect of health on early retirement in relation to the effect of financial incentives. Wang and Shultz^8^ assume on the basis of several studies, that financial motivation may not be a primary driving force for people to keep working, but rather highlight aspects such as the degree of attachment to career jobs and the workers’ commitment to their organizations.

Our findings indicate that with respect to MTW, predictors exhibit their influence differently in different economic subgroups. Leadership quality did not show an influence among the groups with higher incomes, possibly because this group works under relatively privileged working conditions already. The absence of any health effect on MTW among the lowest household net income group indicates the substantially higher relevance and urgency of other factors for this group when it comes to MTW—in line with observations made by Brown and Vickerstaff^22^ that problematic health conditions were subjectively recasted as “tolerable” due to the financial imperatives. This “health‐tolerance” among lower social classes may explain the observation in different large epidemiological studies in Germany that workers in low qualified and manual professions exhibit the by far highest rates of poor health shortly before retirement age,^27^ for example, among women poor health was reported by 67% of all working low‐ and unqualified manual workers aged 60‐64 years, while the respective figures were 31% for managers, 19% for professionals and 6% for engineers (all of same age).^28^ We conclude that finances are an influential domain determining MTW and retirement timing in close interaction with further domains, as proposed by the ‘lidA conceptual model of work, age and employment’.^4^ Yet, the impact of the financial factor also depends on the salience of the work‐retirement transition and on social, cultural and legal framework conditions—which continue to undergo profound changes in many countries.^7^, ^24^

### 4.4 Conceptualizing the interaction of finances and retirement

Considering the fact that financial status is confounded with social class, health and additional employment risks may help to disentangle the complex role of finances for employment participation at higher working age. Radl^5^ distinguishes “involuntary” from “voluntary” early retirement (which may be equivalent to what Meadows means by differentiating “reluctantly retired” from “enthusiastically retired”^12^). When analyzing interview data from eleven Western European countries, Radl found that the social class effects for involuntary early retirement were stronger for workers between 50 and 59 years of age and that they diminish after the age of 60 years.^5^ This may be interpreted as follows: until the age of about 60 years, workers of low occupational class bear higher risks for early exit due to poor health and unemployment, while those who reach the age of about 60 years in employment (“survivors”) remain longer in employment – possibly due to financial hardship.

We now take this age limit of 60 years to differentiate between “very early” (up to age 60) and “early” exit (from 61 to before retirement age) and compare the low‐ and high‐income groups with respect to exit from work and the MTW (Table 3). The cells consider the evidence discussed in this paper including the notion of voluntariness added by Radl.^5^ Thereby we are able to provide a plausible overview explaining, for example, the apparent contradiction that the group of low‐income workers leaves employment very early and yet is motivationally stronger attached to employment than all other income groups.

**TABLE 3:** Conceptual overview of early exit behavior and motivation to keep working among low‐income and high‐income groups at higher working age

	“very early” ‐ up to age 60		“early” ‐ from age 60 to regular retirement age	
	Exit from work	Motivation to keep working	Exit from work	Motivation to keep working
Socioeconomic groups of low financial status	*High exit rates* Because of involuntary exit due to disability and/or unemployment.^5^, ^7^, ^8^, ^9^, ^10^, ^11^	*Very high* Because of current and envisaged financial needs in combination with accumulated employment risks. (own findings)	*Late exit* Due to selection/survivor effect in combination with current and envisaged financial needs.^5^, ^13^, ^24^	*Very high*, Because of current and envisaged financial needs.^12^
Socioeconomic groups of high financial status	*Average* If early exit, then predominantly voluntary, made possible by higher household financial wealth.^3^	*Higher than average* Because of more intrinsically motivating work.^3^	*Late exit* Because of more intrinsically motivating work.^7^	*Higher than average* Because of more intrinsically motivating work.^12^

### 4.5 Strengths and limitations

Among the strengths of this investigation are its large size, which permitted the stratified analysis, and its representativeness for older workers of the German baby boomer generation born in 1959 and 1965. Another strength is the broad range of individual and work predictors available in this interview study. Finally, the age‐homogeneity of the sample may be regarded as a strength, as the issue of work‐retirement transition is highly age dependent. This, however, limits the conclusion to older workers in a similar age range.

A study limitation is the cross‐sectional character of the study, which does not allow for causal conclusions. The following waves of the lidA study will provide the opportunity to assess whether the findings can be confirmed with respect to employment participation instead of MTW. In this study, we used household equivalized net income as an indicator for the financial domain. Lain^7^ emphasizes that research findings may depend to a certain degree on how the individuals’ financial position is measured. So, we cannot exclude that further measures (eg individual income, household or partners’ wealth, financial commitments) might have led to different results. In our study, household income is assessed by an established standardized interview procedure proven to provide best possible validity in self‐report settings. We tried to consider reliability and validity issues by choosing a relative short accounting period (monthly instead of annual) for the assessment of self‐reported income in our study. As Cantillon et al^29^ have found, the annual income indicator performs worse on some measures of data‐quality than the monthly one. We nonetheless are aware that self‐report data, especially on income, always suffer from impression management which poses a limitation to our data. The plausibility of our findings, however, supports our choice of indicator.

Finally, we cannot exclude that attrition has influenced the findings. 13.4% of 3270 participants were lost due to complete case analysis performed in this study. However, the significance of MTW group differences between the five income groups A‐E seems to be robust. Level of significance even increased when the group of 3270 participants was used for analysis (post hoc tests: A to C *P* < .01; A to D *P* < .001; B to D *P* = .060) and it was comparable to the present findings when the total study wave 3 was used for analysis (n = 3364, post hoc tests: A to C *P* < .05; A to D *P* < .01; B to D *P* = .052).

## 5 CONCLUSIONS

In conclusion, in the German employed baby boomer generation (aged 53 and 59 years) those with the lowest income report the highest motivation to work longer—this, in spite of most adverse scores for personal and job indicators. Furthermore, in this group, the MTW is unrelated to physical and mental health, indicating that this comparably high motivation is mainly driven by financial imperatives. This indicates that in Germany, the low‐income group approaching retirement age not only constitutes a risk group with respect to finances, but also to future employment and also personal health. Thus, it requires increased attention by occupational health, research and also by policy which needs to acknowledge potential negative social effects of the regulations effectively extending working lives.

## DISCLOSURES

*Approval of the research protocol*: Design and conduct of the lidA study have been approved by the Ethics Committee of the University of Wuppertal dated from 05/12/2008 and 20/11/2017. *Informed consent*: Participants were fully informed about the aim and procedure of this study prior to giving consent to participate in this study. All procedures performed in studies involving human participants were in accordance with the ethical standards of the institutional and/or national research committee and with the 1964 Helsinki declaration and its later amendments or comparable ethical standards. *Registry and the registration no. of the study/trial*: N/A. *Animal studies*: N/A. *Conflict of interest*: The authors declare that they have no competing interests.

## AUTHORS’ CONTRIBUTIONS

HMH had the idea for the study and developed the study design with AV. AV performed the analyses and wrote the first draft of the article together with HMH who finalised it. ME contributed with her expertise in all steps. All authors critically reviewed and approved the final manuscript.
